# Supplementary material for: Compellingly high SARS-CoV-2 susceptibility of Golden Syrian hamsters suggests multiple zoonotic infections of pet hamsters during the COVID-19 pandemic
Source: Sci Rep. 2022 Sep 5;12:15069. doi: 10.1038/s41598-022-19222-4 (PMC9442591; doi:10.1038/s41598-022-19222-4)
Supplement: Supplementary file 1 — Supplementary Information. [file 41598_2022_19222_MOESM1_ESM.pdf]

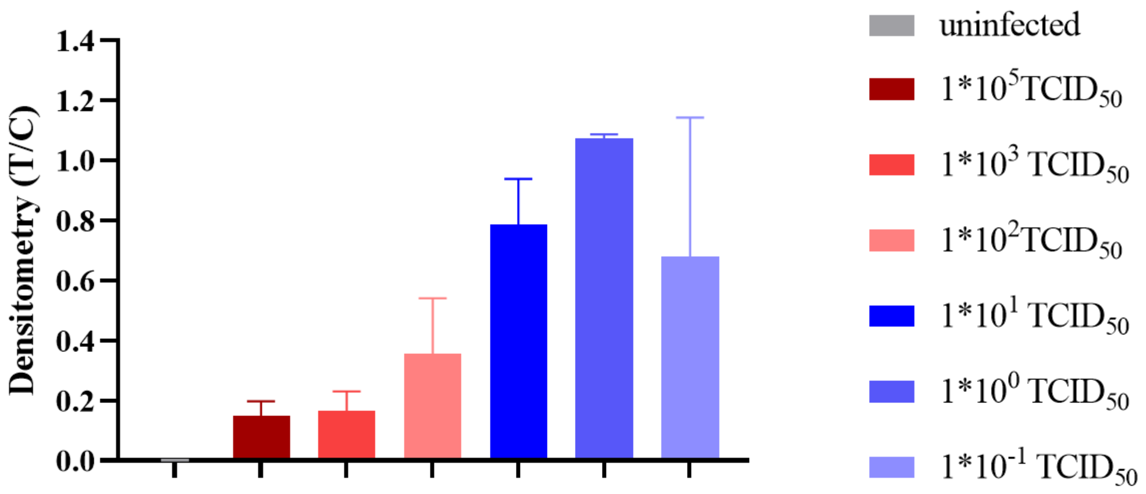

Group	uninfected	$1*10^5$ $TCID_{50}$	$1*10^3$ $TCID_{50}$	$1*10^2$ $TCID_{50}$	$1*10^1$ $TCID_{50}$	$1*10^0$ $TCID_{50}$	$1*10^{-1}$ $TCID_{50}$
N	3	3	3	3	3	3	3
Ct (SD)	-	27,88 (2,68)	33,04 (0)	28,95 (1,65)	27,13 (4,38)	24,37 (1,53)	27,52 (1,13)
TCID50		$< 10^{1,5}$	$< 10^{1,5}$	$< 10^{1,5}$	$10^{1,74}$	$10^{2.56}$	$< 10^{1,5}$
TCID50 SD		0	0	0	$10^{1.51}$	$10^{1.65}$	0

Supplementary Figure S 1. **Analysis of oral swab samples by NowCheck COVID-19 Ag Test (LFD).** Qualitative results of antigen assay after analysis of animals infected with doses of 10^5 to 10^{-1} $TCID_{50}$.

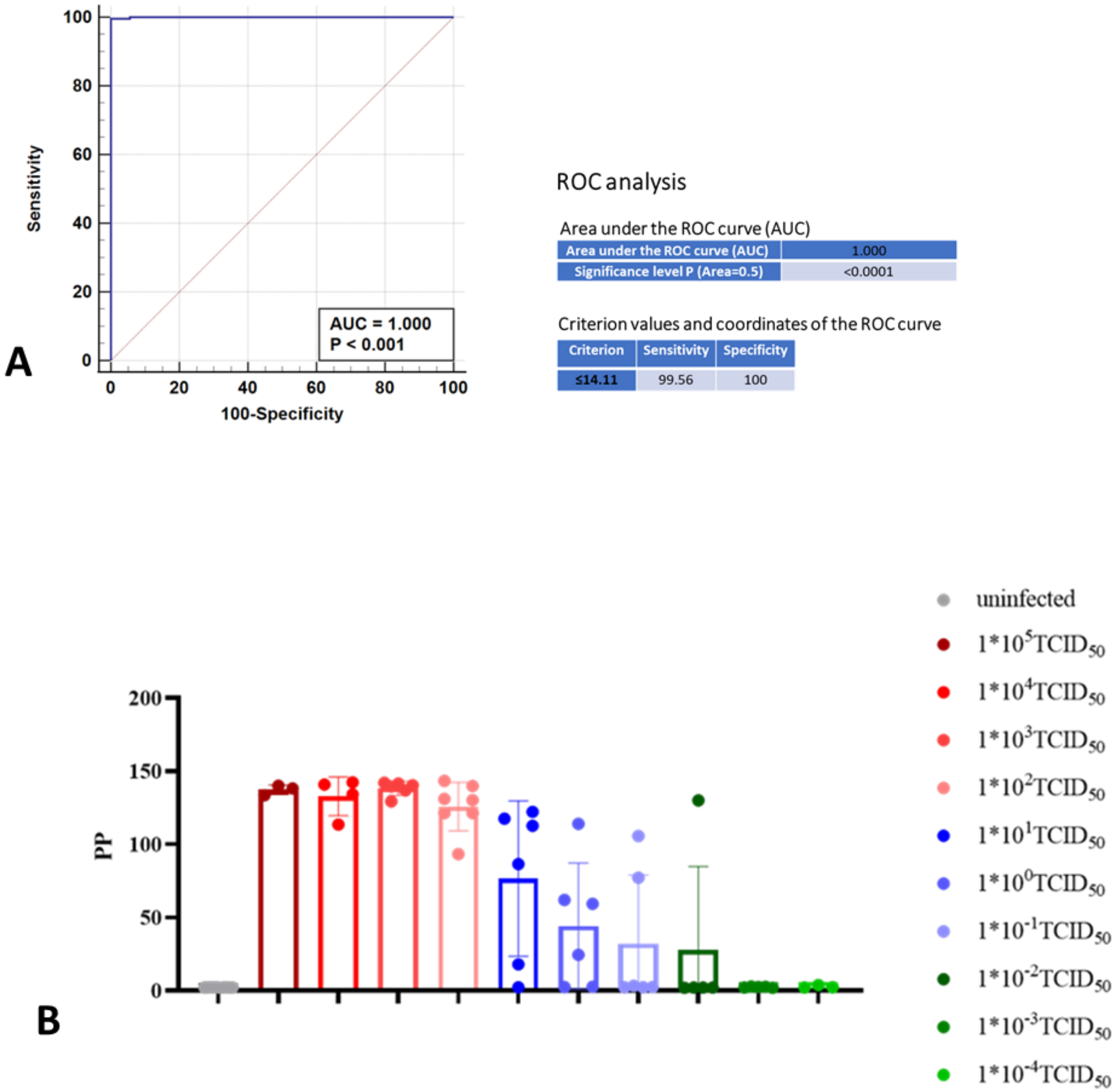

Supplementary Figure S2. SARS-CoV-2 RBD-ELISA results of 280 hamster sera. (A) ROC analysis using 53 negative hamster sera and 227 sera of experimentally SARS-CoV-2 infected hamsters. Diagnostic sensitivity of the SARS-CoV-2 RBD ELISA is 99.56% (95% CI 95.9–99.9) and diagnostic specificity is 100% (95% CI 87.7–100) with AUC 0.999 (p-value <0.01). (B) Dose-dependent development of SARS-CoV-2 specific antibodies within 7-10 days in groups infected

with a dose of $1*10^{-2}$ $TCID_{50}$ or higher, with the variability within the groups increasing at infection doses below $1*10^{2}$ $TCID_{50}$, as shown by the standard error of the mean (SEM).

Supplementary Table S1. Significance levels calculated for the different infection dose groups.

Body weight (%)									Oral swab samples (Real	
**2 dpi**	**3 dpi**	**4 dpi**	**5 dpi**	**6 dpi**	**7 dpi**	**8 dpi**	**9 dpi**	**10 dpi**	**6 dpi**	**8 dpi**
**<0.05***	**<0.05***	**<0.05***	**<0.05***	**<0.05***	**<0.05***	**<0.05***	**<0.05***	**<0.05***	**<0.05***	**<0.05***
Un vs. 10^{4}	Un vs. 10^{4}	Un vs. 10^{5}	Un vs. 10^{5}	Un vs. 10^{5}	Un vs. 10^{5}	Un vs. 10^{2}	Un vs. 10^{2}	Un vs. 10^{2}	10^{5} vs. 10^{-2}	10^{2} vs. 10^{-1}
Un vs. 10^{-3}	Un vs. 10^{2}	Un vs. 10^{4}	Un vs. 10^{4}	Un vs. 10^{4}	Un vs. 10^{4}	Un vs. 10	Un vs. 10	Un vs. 10	10^{4} vs. 10^{-2}	
10^{4} vs. 10^{1}	10^{5} vs. 10^{-1}	Un vs. 10^{3}	Un vs. 10^{3}	Un vs. 10^{3}	Un vs. 10^{3}	10^{2} vs. 10^{-2}	10^{2} vs. 10^{-1}	10^{2} vs. 10^{-1}	10^{3} vs. 10^{-2}	
10^{4} vs. 10	10^{4} vs. 10^{1}	Un vs. 10^{2}	Un vs. 10^{2}	Un vs. 10^{2}	Un vs. 10^{2}	10^{2} vs. 10^{-3}	10^{2} vs. 10^{-2}	10^{2} vs. 10^{-2}	10^{2} vs. 10^{-2}	
10^{4} vs. 10^{-1}	10^{4} vs. 10	10^{5} vs. 10^{-1}	10^{5} vs. 10^{-1}	10^{5} vs. 10^{-1}	10^{5} vs. 10^{-1}	10^{2} vs. 10^{-4}	10^{2} vs. 10^{-3}	10^{2} vs. 10^{-3}	10^{1} vs. 10^{-2}	
10^{3} vs. 10^{-3}	10^{4} vs. 10^{-1}	10^{4} vs. 10^{1}	10^{5} vs. 10^{-2}	10^{5} vs. 10^{-2}	10^{5} vs. 10^{-2}	10 vs. 10^{-2}	10^{2} vs. 10^{-4}	10^{2} vs. 10^{-4}	10 vs. 10^{-2}	
10^{2} vs. 10^{-1}	10^{4}/10^{-2}	10^{4} vs. 10	10^{4} vs. 10^{1}	10^{5} vs. 10^{-3}	10^{4} vs. 10^{-1}	10 vs. 10^{-3}	10 vs. 10^{-2}	10 vs. 10^{-2}	10^{-1} vs. 10^{-2}	
10^{1} vs. 10^{4}	10^{4}/10^{-3}	10^{4} vs. 10^{-1}	10^{4} vs. 10	10^{5} vs. 10^{-4}	10^{4} vs. 10^{-2}	10 vs. 10^{-4}	10 vs. 10^{-3}	10 vs. 10^{-3}	10^{-3} vs. 10^{-2}	
10^{1} vs. 10^{-3}	10^{4}/10^{-4}	10^{4} vs. 10^{-2}	10^{4} vs. 10^{-1}	10^{4} vs. 10^{1}	10^{4} vs. 10^{-3}	10^{-1} vs. 10^{-2}	10 vs. 10^{-4}	10 vs. 10^{-4}		
10 vs. 10^{-3}	10^{3}/10^{-1}	10^{4} vs. 10^{-3}	10^{4} vs. 10^{-2}	10^{4} vs. 10	10^{4} vs. 10^{-4}		10^{-1} vs. 10^{-2}	10^{-1} vs. 10^{-2}		
10^{-1} vs. 10^{-3}	10^{2}/10^{1}	10^{4} vs. 10^{-4}	10^{4} vs. 10^{-3}	10^{4} vs. 10^{-1}	10^{2} vs. 10^{-1}		10^{-1} vs. 10^{-3}	10^{-1} vs. 10^{-3}		
10^{-2} vs. 10^{-3}	10^{2}/10	10^{3} vs. 10^{-1}	10^{4} vs. 10^{-4}	10^{4} vs. 10^{-2}	10^{2} vs. 10^{-2}		10^{-1} vs. 10^{-4}	10^{-1} vs. 10^{-4}		
	10^{2}/10^{-1}	10^{2} vs. 10	10^{3} vs. 10^{-1}	10^{4} vs. 10^{-3}						
	10^{2}/10^{-2}	10^{2} vs. 10^{-1}	10^{2} vs. 10^{-1}	10^{4} vs. 10^{-4}						
		10^{2} vs. 10^{-2}	10^{2} vs. 10^{-2}	10^{3} vs. 10^{-1}						
		10^{2} vs. 10^{-3}	10^{2} vs. 10^{-3}	10^{2} vs. 10^{-1}						
				10^{2} vs. 10^{-2}						
				10^{2} vs. 10^{-3}						

washings (Realtime PCR)		Organs (7 dpi – Realtime PCR)		
**4 dpi**	**7 dpi**	**Nasal conchae**	**Trachea**	**Lung**
**<0.05***	**<0.05***	**<0.05***	**<0.05***	**<0.05***
10^{5} vs. 10^{-2}	10^{2} vs. 10^{-2}	10^{3} vs. 10^{-2}	10^{4} vs. 10^{-3}	10^{5} vs. 10^{-2}
10^{5} vs. 10^{-3}	10^{2} vs. 10^{-3}	10^{1} vs. 10^{-2}	10^{3} vs. 10^{-3}	10^{4} vs. 10^{-2}
10^{5} vs. 10^{-4}	10 vs. 10^{-2}	10 vs. 10^{-2}		10^{3} vs. 10^{0}
10^{4} vs. 10^{-2}	10 vs. 10^{-3}	10^{-1} vs. 10^{-2}		10^{3} vs. 10^{-1}
10^{4} vs. 10^{-3}	10^{-1} vs. 10^{-2}			10^{3} vs. 10^{-2}
10^{4} vs. 10^{-4}	10^{-2} vs. 10^{-3}			10^{3} vs. 10^{-3}
10^{3} vs. 10^{-2}				10^{2} vs. 10^{0}
10^{3} vs. 10^{-3}				10^{2} vs. 10^{-2}
10^{3} vs. 10^{-4}				10^{1} vs. 10^{-2}
10^{2} vs. 10^{-2}				10^{0} vs. 10^{-2}
10^{2} vs. 10^{-3}				10^{-1} vs. 10^{-2}
10^{2} vs. 10^{-4}				10^{-2} vs. 10^{-3}
10^{1} vs. 10^{-2}				
10^{1} vs. 10^{-3}				
10^{1} vs. 10^{-4}				
10 vs. 10^{-2}				
10 vs. 10^{-3}				
10 vs. 10^{-4}				
10^{-1} vs. 10^{-2}				
10^{-2} vs. 10^{-3}				
10^{-3} vs. 10^{-4}				

Supplementary Table S2. PCR detection of SARS-CoV-2 sgRNA. N-gene RNA, standardized quantity (SQ) of N gene copy numbers. as well as $TCID_{50}$ for oral swab, nasal wash and tissue samples. Colors match and represent results from the same hamsters as shown in figures.

Infection dose	Sample	dpi	ct sg RNA	Ct N-gene	SQ N-gene	$TCID_{50}$
1 x 10^1 $TCID_{50}$	nasal wash	2	32.88	29.14	1.60E+03	<10^1.5
1 x 10^1 $TCID_{50}$	nasal wash	2	22.52	21.60	2.83E+05	<10^1.5
1 x 10^1 $TCID_{50}$	nasal wash	2	25.18	22.90	1.16E+05	10^5.25
1 x 10^0 $TCID_{50}$	nasal wash	2	32.87	29.16	1.57E+03	<10^1.5
1 x 10^0 $TCID_{50}$	nasal wash	2	23.19	20.26	7.11E+05	10^5
1 x 10^0 $TCID_{50}$	nasal wash	2	28.53	23.50	7.67E+04	10^3
1 x 10^{-1} $TCID_{50}$	nasal wash	2	27.27	24.42	4.07E+04	10^2.25
1 x 10^{-1} $TCID_{50}$	nasal wash	2	39.87	41.14	4.23E-01	<10^1.5
1 x 10^{-1} $TCID_{50}$	nasal wash	2	27.89	22.69	1.34E+05	10^2.5
1 x 10^{-2} $TCID_{50}$	nasal wash	2	neg	neg	N/A	-
1 x 10^{-2} $TCID_{50}$	nasal wash	2	neg	neg	N/A	-
1 x 10^{-3} $TCID_{50}$	nasal wash	2	18.59	neg	N/A	-
1 x 10^{-3} $TCID_{50}$	nasal wash	2	neg	neg	N/A	-
1 x 10^{-3} $TCID_{50}$	nasal wash	2	neg	neg	N/A	-
1 x 10^1 $TCID_{50}$	nasal wash	4	30.50	23.77	1.51E+04	<10^1.5
1 x 10^1 $TCID_{50}$	nasal wash	4	21.44	16.34	1.89E+06	10^5.5
1 x 10^1 $TCID_{50}$	nasal wash	4	25. Jan	20.16	1.57E+05	10^3.5
1 x 10^0 $TCID_{50}$	nasal wash	4	17. Apr	14.33	6.97E+06	10^6.75
1 x 10^0 $TCID_{50}$	nasal wash	4	16.41	Dez 69	2.03E+07	10^6.25
1 x 10^0 $TCID_{50}$	nasal wash	4	17. Sep	Dez 23	2.73E+07	10^6.5
1 x 10^{-1} $TCID_{50}$	nasal wash	4	18.78	16.16	2.12E+06	10^5.5
1 x 10^{-1} $TCID_{50}$	nasal wash	4	23.43	18.26	5.41E+05	10^5
1 x 10^{-1} $TCID_{50}$	nasal wash	4	23.78	18.57	4.43E+05	10^4.25
1 x 10^{-2} $TCID_{50}$	nasal wash	4	N/A	36.98	2.82E+00	<10^1.5
1 x 10^{-2} $TCID_{50}$	nasal wash	4	37.29	37.41	2.13E+00	10^6
1 x 10^{-3} $TCID_{50}$	nasal wash	4	24.45	24. Apr	1.26E+04	10^2.75
1 x 10^{-3} $TCID_{50}$	nasal wash	4	34.05	29.29	4.18E+02	<10^1.5
1 x 10^{-3} $TCID_{50}$	nasal wash	4	N/A	37.61	1.45E+00	<10^1.5
1 x 10^{-2} $TCID_{50}$	oral swab	6	.	35.19	9.34E+00	<10^1.5
1 x 10^{-2} $TCID_{50}$	oral swab	6	N/A	36.89	2.98E+00	<10^1.5
1 x 10^{-3} $TCID_{50}$	oral swab	6	28.56	23.77	1.99E+04	10^2
1 x 10^{-3} $TCID_{50}$	oral swab	6	29.24	24.70	1.07E+04	<10^1.5
1 x 10^{-3} $TCID_{50}$	oral swab	6	32.73	28.75	7.03E+02	<10^1.5

1 x 10^{2} TCID$_{50}$	nasal conchae	7	19.67	14.57	1.06E+07	10^3.5
1 x 10^{2} TCID$_{50}$	trachea	7	34.16	29.98	2.56E+02	<10^1.5
1 x 10^{2} TCID$_{50}$	lung	7	21.71	19.98	2.67E+05	10^2
1 x 10^{2} TCID$_{50}$	nasal conchae	7	22.64	18.17	9.14E+05	10^1.75
1 x 10^{2} TCID$_{50}$	trachea	7	35.18	31.72	8.93E+01	<10^1.5
1 x 10^{2} TCID$_{50}$	lung	7	25.95	23.74	2.06E+04	<10^1.5
1 x 10^{2} TCID$_{50}$	nasal conchae	7	23.93	19.86	2.89E+05	<10^1.5
1 x 10^{2} TCID$_{50}$	trachea	7	36.89	33.16	3.03E+01	<10^1.5
1 x 10^{2} TCID$_{50}$	lung	7	27.55	26.65	2.82E+03	<10^1.5
1 x 10^{1} TCID$_{50}$	nasal chonchae	7	22.59	17.19	1.79E+06	10^3
1 x 10^{1} TCID$_{50}$	trachea	7	26.23	19.31	4.20E+05	<10^1.5
1 x 10^{1} TCID$_{50}$	lung	7	21.77	20.34	2.09E+05	<10^1.5
1 x 10^{1} TCID$_{50}$	nasal conchae	7	24.62	19.71	3.21E+05	<10^1.5
1 x 10^{1} TCID$_{50}$	trachea	7	29.13	25.34	6.93E+03	<10^1.5
1 x 10^{1} TCID$_{50}$	lung	7	29.50	27.15	2.02E+03	<10^1.5
1 x 10^{1} TCID$_{50}$	nasal conchae	7	17.99	15.44	5.89E+06	10^8.5
1 x 10^{1} TCID$_{50}$	trachea	7	30.29	25.87	4.81E+03	<10^1.5
1 x 10^{1} TCID$_{50}$	lung	7	21.54	20. Jan	2.62E+05	10^2
1 x 10^{0} TCID$_{50}$	nasal conchae	7	19.89	17.28	1.68E+06	10^7.5
1 x 10^{0} TCID$_{50}$	trachea	7	31.63	39.35	4.91E-01	<10^1.5
1 x 10^{0} TCID$_{50}$	lung	7	18.74	16.18	3.55E+06	10^4.5
1 x 10^{0} TCID$_{50}$	nasal conchae	7	25.91	22.14	6.13E+04	10^3.25
1 x 10^{0} TCID$_{50}$	trachea	7	30.47	N/A	neg	neg
1 x 10^{0} TCID$_{50}$	lung	7	19.47	15.70	2.47E+06	10^3.75
1 x 10^{0} TCID$_{50}$	nasal conchae	7	20.50	15. Jan	3.93E+06	10^4.25
1 x 10^{0} TCID$_{50}$	trachea	7	33.35	30.46	1.86E+02	10^2.5
1 x 10^{0} TCID$_{50}$	lung	7	19.73	17.13	9.36E+05	10^3.5
1 x 10^{-1} TCID$_{50}$	nasal conchae	7	21.59	15.89	2.17E+06	-
1 x 10^{-1} TCID$_{50}$	trachea	7	39.46	36.75	1.58E+00	<10^1.5
1 x 10^{-1} TCID$_{50}$	lung	7	22.31	18.70	3.24E+05	10^2.25
1 x 10^{-1} TCID$_{50}$	nasal conchae	7	22. Mai	17.35	8.07E+05	10^2.5
1 x 10^{-1} TCID$_{50}$	trachea	7	25.20	19.50	1.88E+05	<10^1.5
1 x 10^{-1} TCID$_{50}$	lung	7	18.42	15. Jun	3.81E+06	10^7.25
1 x 10^{-1} TCID$_{50}$	nasal conchae	7	29.21	23.35	1.38E+04	<10^1.5
1 x 10^{-1} TCID$_{50}$	trachea	7	32.33	29.88	2.75E+02	10^1.75
1 x 10^{-1} TCID$_{50}$	lung	7	21.59	18.80	3.03E+05	10^4.75
1 x 10^{-2} TCID$_{50}$	nasal conchae	7	22.77	17.54	7.09E+05	10^6.5
1 x 10^{-2} TCID$_{50}$	trachea	7	27.48	22.47	2.52E+04	10^3

1 x 10^{-2} $TCID_{50}$	lung	7	40.80	36.89	1.44E+00	<10^1.5
1 x 10^{-2} $TCID_{50}$	nasal conchae	7	41.48	36.45	1.94E+00	<10^1.5
1 x 10^{-2} $TCID_{50}$	trachea	7	N/A	N/A	N/A	N/A
1 x 10^{-2} $TCID_{50}$	lung	7	N/A	36.39	2.02E+00	<10^1.5
1 x 10^{-3} $TCID_{50}$	nasal conchae	7	20. Dez	15.27	3.30E+06	10^5
1 x 10^{-3} $TCID_{50}$	trachea	7	24.14	18.57	3.54E+05	10^3
1 x 10^{-3} $TCID_{50}$	lung	7	21. Nov	17.52	7.19E+05	<10^1.5
1 x 10^{-3} $TCID_{50}$	nasal conchae	7	26. Okt	19.75	1.59E+05	10^3
1 x 10^{-3} $TCID_{50}$	trachea	7	26.43	19.98	1.36E+05	10^2.75
1 x 10^{-3} $TCID_{50}$	lunge	7	19.13	15.14	3.61E+06	<10^1.5
1 x 10^{-3} $TCID_{50}$	nasal conchae	7	22. Sep	16. Aug	1.91E+06	10^6
1 x 10^{-3} $TCID_{50}$	trachea	7	25.74	19.43	1.98E+05	10^3.75
1 x 10^{-3} $TCID_{50}$	lung	7	21.31	17.52	7.21E+05	<10^1.5
